# Algal Phycocolloids: Bioactivities and Pharmaceutical Applications

**DOI:** 10.3390/md21070384

**Published:** 2023-06-28

**Authors:** Silvia Lomartire, Ana M. M. Gonçalves

**Affiliations:** 1 University of Coimbra, MARE-Marine and Environmental Sciences Centre/ARNET-Aquatic Research Network, Department of Life Sciences, Calçada Martim de Freitas, 3000-456 Coimbra, Portugal; silvia.lomartire@student.uc.pt; 2 Department of Biology and CESAM, University of Aveiro, 3810-193 Aveiro, Portugal

**Keywords:** seaweed, polysaccharides, carrageenan, alginate, agar, therapeutic applications, new drug delivery system

## Abstract

Seaweeds are abundant sources of diverse bioactive compounds with various properties and mechanisms of action. These compounds offer protective effects, high nutritional value, and numerous health benefits. Seaweeds are versatile natural sources of metabolites applicable in the production of healthy food, pharmaceuticals, cosmetics, and fertilizers. Their biological compounds make them promising sources for biotechnological applications. In nature, hydrocolloids are substances which form a gel in the presence of water. They are employed as gelling agents in food, coatings and dressings in pharmaceuticals, stabilizers in biotechnology, and ingredients in cosmetics. Seaweed hydrocolloids are identified in carrageenan, alginate, and agar. Carrageenan has gained significant attention in pharmaceutical formulations and exhibits diverse pharmaceutical properties. Incorporating carrageenan and natural polymers such as chitosan, starch, cellulose, chitin, and alginate. It holds promise for creating biodegradable materials with biomedical applications. Alginate, a natural polysaccharide, is highly valued for wound dressings due to its unique characteristics, including low toxicity, biodegradability, hydrogel formation, prevention of bacterial infections, and maintenance of a moist environment. Agar is widely used in the biomedical field. This review focuses on analysing the therapeutic applications of carrageenan, alginate, and agar based on research highlighting their potential in developing innovative drug delivery systems using seaweed phycocolloids.

## 1. Introduction

The demand for healthy and natural products from health-conscious consumers has led to significant growth in the global hydrocolloids market. Hydrocolloids find applications in various industries such as oil, food, paper, paint, textiles, and pharmaceuticals. Their diverse range of functions plays a crucial role in driving the market forward. The primary types of hydrocolloids include gelatin, pectin, xanthan gum, and guar gum.

Among these, gelatin holds the largest share in the food hydrocolloids market due to its extensive use as a gelling agent in confectionary, meat, poultry, and dairy products. Hydrocolloids can be classified as natural, semisynthetic, or synthetic, depending on their origin. Natural hydrocolloids are hydrophilic biopolymers derived from plants, animals, or microbes. Plant-derived hydrocolloids are primarily used to stabilize oil-in-water emulsions, while animal-derived hydrocolloids tend to form water-in-oil emulsions.

However, animal-derived hydrocolloids can potentially cause allergies and are prone to microbial growth and rancidity. Semisynthetic hydrocolloids are synthesized by modifying naturally occurring hydrocolloids. Examples of semisynthetic hydrocolloids include starch and cellulose derivatives such as methylcellulose (MC), carboxymethylcellulose (CMC), hydroxypropyl methylcellulose (HPMC), microcrystalline cellulose (MCC), acetylated starch (AS), phosphorylated starch (PS), and hydroxypropylated starch (HPS). These semisynthetic hydrocolloids exhibit stronger emulsifying properties, are nontoxic, and are less prone to microbial growth. On the other hand, synthetic hydrocolloids are completely synthesized in industries using petroleum-derived base materials. They are the most potent emulsifiers and do not support microbial growth, but their costs can be prohibitive. Synthetic hydrocolloids are primarily used as oil-in-water emulsifiers. However, semisynthetic hydrocolloids are generally preferred over purely synthetic gums [1,2].

Various hydrocolloids, including guar gum, carrageenan, xanthan gum, gum arabic, pectin, and others, have demonstrated potential in preserving and safeguarding pharmaceutical drugs from external stresses [3]. Guar gum possesses inherent gelling properties, pH-responsive behaviour due to its ionic groups, and vulnerability to bacterial and enzyme degradation in the large intestine. These characteristics make it an excellent choice as a carrier for drug delivery targeted specifically to the colon [4]. This water-soluble polysaccharide has been utilized in various forms such as matrix tablets, compression-coated tablets, nanoparticles, and hydrogels. Additionally, significant research highlights the application of guar gum in protein delivery, antihypertensive medications, and transdermal drug delivery systems [4,5]. However, a notable limitation of its usage stems from its high swelling properties, which can result in the rapid release of loaded drug molecules [6]. Hydrocolloids can be derived from both renewable and non-renewable resources. However, there is a growing preference for renewable hydrocolloids due to economic and ecological considerations. In response to the increasing consumer demand for all-natural products, the goal is to replace nonrenewable and synthetic hydrocolloids with renewable alternatives in various industrial applications. This drives the search for novel natural hydrocolloids that can offer unique features for specific purposes [7].

In the food and pharmaceutical industries, natural hydrocolloids are highly favoured over semisynthetic and synthetic hydrocolloids. This preference is due to their numerous distinct advantages, which contribute to enhancing the stability, functionality, quality, safety, and nutritional value of various products. Natural hydrocolloids possess several notable benefits compared to their counterparts, including being extracted from renewable sources, readily available and easy to work with, biocompatible, nontoxic, capable of physical and chemical modification, environmentally friendly, cost-effective, and widely accepted by the public due to the multitude of health benefits they offer [8].

Therefore, there is a growing interest in seaweeds as sources of hydrocolloids. The historical utilization of seaweeds, also referred to as macroalgae, for medicinal applications can be traced back to Asian countries. These cultures explored the remarkable benefits of seaweeds and incorporated them as alternative healing methods. Seaweeds are categorized into three groups: brown algae (Ochrophyta, class Phaeophyceae), red algae (Rhodophyta), and green algae (Chlorophyta). Every division contains an assortment of bioactive substances displaying diverse properties and mechanisms of action. In addition to their protective actions, seaweed metabolites offer a high nutritional content and numerous health advantages [9,10]. Seaweeds have proven to serve as versatile natural stores of different metabolites [11]. Multiple research studies showcase that bioactive compounds derived from seaweeds have applications across various sectors, including the production of wholesome edibles [12], pharmaceutical formulations [13], and cosmetic commodities [14]. Algal compounds are harnessed for the manufacturing of food products, animal feed, beauty items, and fertilizers [15,16,17,18,19].

According to the findings from the literature, seaweeds have a low fat and lipid content (polyunsaturated fatty acids) but are rich in polyphenols, carbohydrates, proteins, trace elements, nutrients, and pigments [20,21,22]. While the chemical makeup of seaweeds is not as extensively understood as that of land plants, it has been found that specific organic compounds are exclusively synthesized by marine algae [23].

Hydrocolloids derived from seaweeds, such as agar, carrageenan (abundant in Rhodophyta), and alginates (abundant in Phaeophyceae), are extensively harvested and employed across multiple sectors. They serve as gelling agents in food and dressings, coatings in pharmaceutical products, stabilizers in biotechnology, and ingredients in cosmetics such as moisturizers, body lotions, hair cleansers, and dental paste [24,25]. By exploring their beneficial qualities, there is potential for the creation of targeted functional food products tailored to different needs and suitable for medical purposes [26]. Carrageenan has gained significant interest and its usage in pharmaceutical formulations has increased. It has been incorporated into recognized pharmacopoeias such as the United States Pharmacopeia 35-National Formulary 30 S1 (USP35-NF30 S1), British Pharmacopoeia 2012 (BP2012), and European Pharmacopoeia 7.0 (EP7.0), indicating its potential as a pharmaceutical excipient and a promising future [27].

Carrageenan has demonstrated various pharmaceutical properties such as antiviral and antimicrobial properties [28,29,30,31,32], but also anticoagulant effects [33,34], antidiabetic [35,36] and antioxidant activity [37,38,39], and more. There is a growing interest in utilizing mixtures and combinations of carrageenan with natural polymers such as chitosan, starch, cellulose, chitin, and alginate, which are explored to create biodegradable materials with favourable characteristics for use in biomedical applications. These combinations have shown significant potential in various biomedical purposes, including drug delivery and tissue engineering [40].

Alginate, a polysaccharide found in nature, is highly valued for its applications in manufacturing wound dressings due to its unique characteristics. These include low toxicity, biodegradability, cost-effectiveness, the ability to form hydrogels, prevention of bacterial infections, and the ability to maintain a moist environment [41,42].

Agar or agar-agar, a unique naturally occurring polymer, is increasingly preferred over synthetic polymers and is being explored as an alternative raw material for medicinal applications. It holds significant appeal in the pharmaceutical sector due to its exceptional inherent qualities, particularly the strong gel it forms. Agar-agar has been utilized in the development of injectable and phase-changeable composite hydrogels for treating cancers with chemo and photothermal therapy. These composite hydrogels can effectively load and release chemotherapeutics and antibiotics. Additionally, an agar-based nanocomposite film has demonstrated effectiveness in inhibiting the growth of *Listeria monocytogenes* [43]. In the pharmaceutical industry, the use of agar and polysaccharide blends is also gaining popularity. Agar-agar primarily serves as a gelation, stabilization, and thickening agent in pharmaceuticals. Moreover, it is commonly employed for purgative purposes and as a surgical aid. Researchers have dedicated efforts to creating agar-based products such as composite hydrogels, nanocomposite films, and other materials specifically to be applied in the field of pharmacology [43].

The combination of agar molecules with the lowest concentrations of charge results in the formation of agarose exhibits excellent gel-forming ability. Agar, on the other hand, is present in varying amounts with complexed molecules and different levels of charged groups. The capacity of agar to withstand hydrolysis is crucial for its application in bacteriology. When preparing culture media, agar’s strong gel-strengthening property and lack of cations with hysteresis contribute to the production of high-quality solid microbial cultures. Agarose finds a wide range of uses in biotechnology, and the increasing number of innovative applications is expected to drive the demand for high-quality agarose in the field [44]. Agar derivatives have also found applications in dentistry and biotechnology, such as dental prosthesis, material shaping, and plant culture tissues [45].

The objective of the present review is to evaluate the possibility of seaweed hydrocolloids for the development of new drug delivery systems. To make it possible, investigations are needed to assess the mechanical and chemical characteristics of carrageenan, alginate, and agar and their therapeutic properties.

## 2. Seaweeds Phycocolloids

### 2.1. Carrageenan

**Figure 1:**
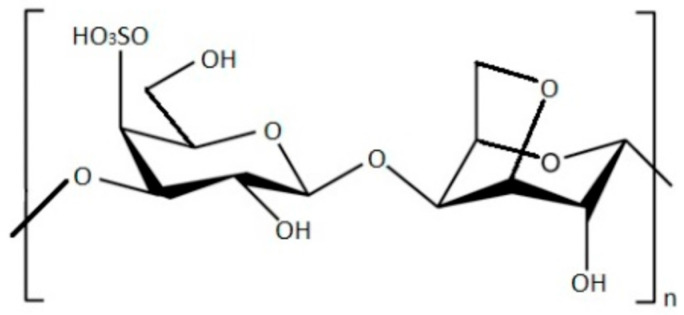
Chemical structure of κ-carrageenan.

Red algae contain a type of polysaccharides called carrageenans [46]. These carrageenans consist of galactans, which can form a gel in aqueous or milk solutions. These compounds are extensively employed in the food, cosmetic, and pharmaceutical sectors. As a result, extracts from red algae containing carrageenans are commercially utilized [47,48]. Carrageenan, illustrated in Figure 1, is a linear polysaccharide composed of sulphated or nonsulphated galactose units linked together through α-1,3-glycosidic and β-1,4-galactose bonds [49]. These natural polysaccharides consist of a mixture of sulphated linear galactans. The structural units are composed of disaccharides, specifically α-(1→4)-linked d-galactopyranose (D) residue or 3,6-anhydrogalactopyranose (DA), and β-(1→3)-linked d-galactopyranose (G) residue. The sulphate groups are bound by covalent bonds to the galactose atoms C-2, C-4, or C-6 through ether bonds [46].

Carrageenans can be classified into various types (κ-carrageenan, ι-carrageenan, λ-carrageenan, γ-carrageenan, ν-carrageenan, ξ-carrageenan, θ-carrageenan, and µ-carrageenan) based on the position of the sulphate group attached to the galactose unit. In nature, carrageenans are predominantly hybrid, leading to variations in their properties depending on the specific bonded sulphate group [50]. These carrageenans are typically categorized into three structural configurations (κ-, ɩ-, and ʎ-) according to the quantity of sulphated groups attached to the galactose unit; the presence, chemical position, and organization of these groups govern the function and bioactive properties of carrageenan [51].

Different species yield distinct types of carrageenans. κ-carrageenans, readily accessible in the market, are extracted from *Kappaphycus alvarezii* through a heat extraction method. In contrast, λ-carrageenans are typically derived from red algae species found in the *Gigartina* or *Chondrus* genera through drum drying or ethanol precipitation methods [52]. These carrageenans are found in various families such as Solieriaceae, Rhabdoniaceae, Phyllophoraceae, Gigartinaceae, Rhodophilidaceae, and Thichocarpaceae. Among these, eight carrageenan sources are exclusive to the Japanese Sea (East Sea), with *Chondrus pinnulatus*, *Chondrus armatus*, *Chondrus yendoi*, *Mastocarpus pacificus*, and *Mazzaella hemisphaerica* belonging to the Gigartinaceae and Solieriaceae families. In addition to Gigartinaceae, significant quantities of carrageenan can be found in algae belonging to the Phillophoraceae and Thichocarpaceae families, whose species are extensively distributed in the seas of the Far East [46].

In contrast, ɩ-carrageenan is primarily derived from *Eucheuma denticulatum* (commonly known as “spinosum”), and it imparts a soft and weak gel consistency. Lastly, ʎ-carrageenan is obtained from various species of the *Gigartina* and *Chondrus* genera [53].

Carrageenans find extensive use in the food industry due to their ability to gel, thicken, and stabilize food products [54]. The commercial forms of ʎ-, κ-, and ɩ-carrageenans have been approved as food additives by regulatory bodies such as the Food and Drug Administration (FDA) and the European Food Safety Agency (EFSA) [55]. Over the past few decades, the medical field has also explored the biological potential of carrageenan, yielding positive results. It has been determined that these compounds exhibit anticoagulant and antithrombotic activity [56], antiviral properties [57], antitumoral effects [58], and antioxidant properties [39].

Water solubility is characteristic of all carrageenan variants, albeit their solubility in aqueous solutions is subject to influence from factors such as temperature, pH, ionic strength, and the presence of cations. The hydrophilic nature of carrageenans stems from the sulphate and hydroxyl groups, while their hydrophobic characteristics mainly arise from the 3,6-anhydro-α-D-galactopyranose units [54].

The hydrophobicity of carrageenan presents a drawback in the production of water-resistant packaging. However, one potential solution to enhance the properties of carrageenan is to combine it with hydrophobic compounds to reinforce the material’s matrix. This approach could result in ecofriendly and cost-effective packaging materials with improved strength, as well as potential therapeutic applications [59].

Saluri et al. [34] conducted a study to investigate the anticoagulant and antioxidant activity of λ-carrageenan, which was alkali treated to obtain θ-carrageenan. They also compared the molecular weights and biochemical parameters of different carrageenan types and examined their biological activities. Initially, λ-carrageenan exhibited a significantly larger molecular weight, approximately five times greater than that of θ-carrageenan. After an extensive 72 h autohydrolysis process, the molecular weight of λ-carrageenan was four times higher than that of θ-carrageenan. The molecular weight range of λ-carrageenan samples was between 3100 and 4.7 kDa, while that of θ-carrageenan samples ranged from 630 to 1.1 kDa. The degradation of polysaccharides displayed remarkable dynamics, allowing for the accurate prediction of the degree of degradation based on the hydrolysis duration [34]. The purity of nondegraded λ- and θ-carrageenan samples was confirmed through infrared spectrometry (FTIR) measurements. Significant changes due to degradation were observed in the region ranging from approximately 1200 to 1000 cm^−1^. Nondegraded carrageenans exhibit distinct features that could be potentially useful for distinguishing between the two polysaccharides. Specifically, λ-carrageenan shows a shouldered absorption maximum in the range of approximately 1100 cm^−1^ to 1000 cm^−1^, while θ-carrageenan displays two well-separated signals. Both spectra demonstrate a prevalent signal at approximately 1220 cm^−1^, which is characteristic of sulphated polysaccharides and can be attributed to sulphate esters. The intensity of the sulphate ester signal decreases with degradation, while new signals emerge around 1100 cm^−1^, indicating detached sulfate groups, particularly evident after 48 h of degradation for λ-carrageenan and 12 h of degradation for θ-carrageenan samples. The primary structural differences between carrageenans can be identified in the spectral region from approximately 750 to 1000 cm^−1^. θ-carrageenan spectra display narrow absorptions at 832 cm^−1^, corresponding to the vibration of C–O–S in 3-linked β-D-galactopyranose-2-sulfate. In contrast, λ-carrageenan spectra exhibit broader signals starting at 819 cm^−1^, attributed to C–O–S in both C-2 and C-6. Vibrations at 806 cm^−1^ and 901 cm^−1^ might be attributed to C-2 in anhydrogalactose, and a sharp signal at 934 cm^−1^ has been identified as an anhydro bridge in anhydrogalactose, which is detectable in θ-carrageenan spectra but not in λ-carrageenan spectra. Signals in the range of 576–612 cm^−1^, arising from bending vibrations of O–S–O, were present in all spectra. C–O axial deformation can be observed as signals at approximately 1155 cm^−1^ for both carrageenans. Additionally, weak signals at 761 and 771 cm^−1^ reveal skeletal bending of the galactopyranose ring [34].

It is important to highlight that the activity of λ-carrageenan samples showed a gradual decrease as the molecular weight decreased, while the effect of molecular weight decrease on θ-carrageenan activity was mainly observed in the short-term autohydrolyzed samples, with the low molecular weight samples maintaining a relatively constant level of activity. The activity of λ-carrageenans experienced significant drops in the molecular weight ranges of 1200 to 280 kDa, 150 to 73 kDa, and from 47 to 23 kDa.

Analysis revealed that λ-carrageenan samples exhibited approximately twice the anticoagulant activity compared to θ-carrageenan preparations. In comparison to the control heparin, high molecular weight λ-carrageenan samples demonstrated nearly two and a half times lower anticoagulant activity, while high molecular weight θ-carrageenan samples exhibited five times lower anticoagulant activity. A notable decrease in activity for λ-carrageenan samples occurred with a molecular weight change from 150 to 73 kDa. The activity of θ-carrageenan reached a plateau from 27 kDa downwards. The FRAP assay results showed an increase in antioxidant activity for degraded samples, with a plateau observed around 15 kDa for θ-carrageenan and 280 kDa for λ-carrageenan samples. On average, θ-carrageenan samples displayed three times higher antioxidant activity compared to λ-carrageenan samples. The FC assay indicated a steep increase in antioxidant activity with decreasing molecular weight for θ-carrageenan, such as the FRAP assay.

This may be attributed to changes in the polysaccharide chain, as indicated by the FTIR-ATR measurements. Polysaccharide degradation had a minimal effect on the antioxidant activity of λ-carrageenan samples in FC tests.

Regarding antioxidant activity, the results indicated that θ-carrageenan samples exhibited greater activity, especially when the samples were degraded. Similar findings have been reported in previous research [60,61] comparing antioxidant activity to changes in molecular weight for other biopolymers.

### 2.2. Agar

**Figure 2:**
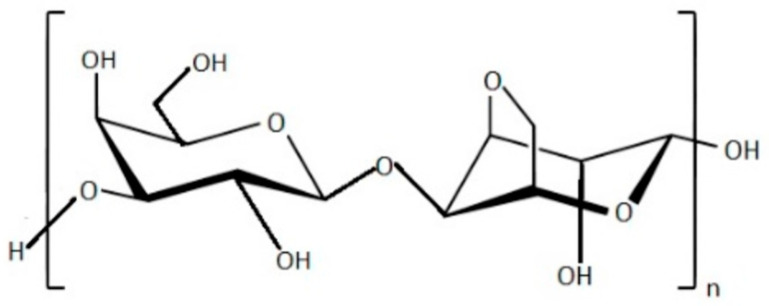
Chemical structure of agarose polymer.

Agar has a chemical structure distinguished by recurring units of D-galactose and 3,6-anhydro-L-galactose, with minor deviations and a low level of sulfate esters (Figure 2); carrageenan comprises two groups of polysaccharides: agarose, an uncharged polysaccharide, and agaropectin, a simplified term for the charged polysaccharide [62,63,64]. Agarose is responsible for agar’s ability to form gels, making it a valuable ingredient in skincare, herbal medicine, and pharmaceuticals. Additionally, it has exceptional film properties [65].

Agar is a term utilized to describe a blend of gelling polysaccharides composed of d-galactose and l-galactose [66]. This combination is synthesized within the cellular wall matrix of red seaweeds and maintains a gel-like structure at room temperature [67]. The specific polysaccharide, known as agarose (Figure 2), is characterized by repeating units of d-galactose and 3,6-anhydro-l-galactose linked together by β-1,3- and α-1,4-glycosidic bonds. Agarose makes up to 70% of the total polysaccharide content in agar [68].

Due to their functions as stabilizers, emulsifiers, and thickeners, agar is widely utilized in the commercial food processing sector. These additives are commonly found in gel-based food products such as desserts, preserves, jellies and baked goods. Agar in gel form typically exhibits a firm and transparent texture, but its strength can be enhanced by incorporating sugars [69]. One advantage of agar is its low hygroscopic property, which is beneficial for packaging production. Additionally, agar films are biologically nonreactive and exhibit a propensity to interact with different bioactive compounds and/or plasticizers, thereby facilitating the production of elastic and soft gel formations [70,71,72].

Agar and other polysaccharides play a crucial role in providing protection to algae against pathogens, maintaining cellular ionic balance, and safeguarding them from extreme conditions such as salinity, pH, temperature variations, and desiccation [73,74].

Furthermore, different types of agars are utilized in various industries, including food, pharmaceuticals, cosmetics, medicine, and biotechnology, such as phycocolloids. The global production of agar has seen a significant increase, from 6800 tons (USD 82.2 million) in 2002 to 9600 tons (USD 173 million) in 2009. The main sources of agar for industrial purposes are *Gracilaria* spp. (80%) and *Gelidium* spp. (20%), with *Gracilaria* spp. known for having the highest degree of sulfation [69,75]. Recently, there has been growing interest in *Gracilaria* spp. agars characterized by weaker gel consistency, due to the decline in *Gelidium* spp. stocks and the successful cultivation of *Gracilaria* [76,77].

Agar, extracted from red seaweeds with a molecular weight range of 100–30,000 kDa [78], has been studied for its various polysaccharide fractions. Among these fractions, the 3.2 kDa fraction isolated from the red seaweed *Pyropia yezoensis* was found to be effective in stress protection [79]. In the work conducted by Martínez-Sanza et al., agar-based extracts from the seaweed *Gelidium sesquipedale* were produced and characterized using simple protocols involving hot water and sonication treatments. The combination of sonication with hot water treatment reduced the extraction time by fourfold without significantly affecting the extraction yield (approximately 10–12%) or the properties of the extracts. These extracts, in addition to agar, contained proteins, polyphenols, and minerals, which contributed to their high antioxidant capacity and resulted in the formation of brownish, softer gels. An alkali pretreatment was also applied to obtain nearly pure agar extracts with higher molecular weights and crystallinities compared to commercial agar, resulting in stiffer gels. The production of agar-based extracts from *G. sesquipedale* involved the use of hot water treatment (HW) and a combined heating-sonication method (HW-US), achieving extraction yields of approximately 10–12%. The combined treatment reduced the total extraction time by fourfold without significantly affecting the extraction yield. Additionally, an alkali pretreatment using NaOH with HW and NaOH with HW-US was evaluated to remove impurities prior to extraction, but it resulted in lower extraction yields of approximately 2–3%. While the NaOH + HW and NaOH + HW-US extracts mainly consisted of agar, the HW and HW-US extracts contained additional compounds such as proteins, polyphenols, and mineral substances, which contributed to their high antioxidant capacity, surpassing that of extracts from other *Gelidium* species.

These unpurified HW and HW-US extracts produced brownish gels with lower gel strength compared to commercial agar, making them suitable as texture modifiers or thickening agents in food-related applications. The simplified extraction protocols used in the production of unpurified agar-based extracts from the red seaweed *G. sesquipedale*, as described by Marta Martínez-Sanza et al., demonstrated the efficiency of the combined heat and sonication method in generating cost-effective agar-based extracts with potential applications in the food industry. However, the alkali treatment resulted in lower extraction yields (approximately 2–3%) due to the partial digestion of agar. These findings highlight the potential of these extracts and their suitability for various applications in the food industry [80]. Nevertheless, the incomplete breakdown of agar caused by the alkali resulted in diminished extraction yields (approximately 2–3%). These findings demonstrate the effectiveness of the combined heat and sonication technique in producing economically viable agar-derived extracts that hold promise for various applications in the food industry [80].

### 2.3. Alginate

**Figure 3:**
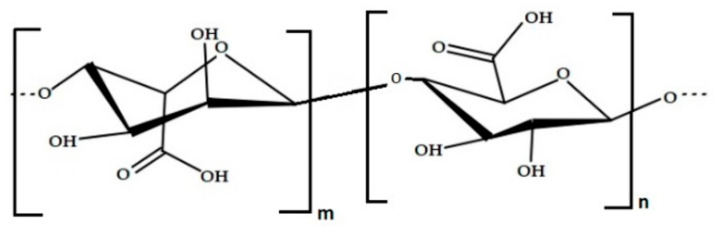
Chemical structure of alginic acid.

The key polysaccharide in this context is alginic acid, also known as algin or alginate (Figure 3). These polysaccharides can be derived from the cell walls of brown algae, including species such as *Macrocystis pyrifera*, *Laminaria hyperborea*, *Ascophyllum nodosum*, as well as various bacterial strains [81]. Alginate, derived from alginic acid and its derivatives and salts [54,82], accounts for 10% to 40% of the dry weight of untreated algae and comprises 30–60% of the total sugars in brown seaweeds [83]. Alginates are anionic linear polysaccharides present in significant amounts in brown seaweeds, constituting up to 40% of the dry weight, and they have been acknowledged for their capacity to create edible films.

These alginates are composed of polymers of alginic acid, with monomer units of β-D-mannuronic acid (M) and α-L-guluronic acid (G) joined by 1,4 linkages [84,85].

The physicochemical and mechanical properties of alginate gels differ based on the M/G ratio and the length of the structure. A higher content of guluronic acid contributes to stronger gelling characteristics and the formation of more elastic gels. On the other hand, lower M/G ratios yield sturdy and brittle gels that demonstrate excellent heat stability but may exhibit syneresis during the freeze–thaw cycle [54,82]. With its remarkable stabilizing and thickening abilities, alginate is extensively utilized in various food and medical applications [54,82,86].

Due to their high hydrophilicity, alginates require the incorporation of additional components within the matrix to enhance water resistance. Furthermore, the presence of ions impacts the solubility of alginates, and the formation of gels relies on the type of bonds formed with cations [54,82]. Introducing calcium into the alginate matrix enhances stability and resilience, offering potential for the creation of biodegradable materials with antimicrobial properties and nontoxic packaging [86,87].

Due to their high hydrophilicity, alginates require the incorporation of other components into the matrix to enhance their resistance when in contact with water. Furthermore, the solubility of alginates is influenced by the presence of ions, and the formation of gels depends on the specific bonds formed with cations [54,82]. The introduction of calcium into the alginate matrix enhances the stability and resilience of the membrane, showcasing the potential for the development of biodegradable materials with antimicrobial properties and nontoxic applications in medical support [86,87].

Alginates possess favourable attributes such as biodegradability, biocompatibility, nontoxic behaviour, and affordability, making them highly suitable for various biological applications [88]. Alginates can be sourced from a variety of seaweed species, including *Ascophyllum* spp., *Durvillaea* spp., *Laminaria* spp., *Lessonia* spp., *Macrocystis* spp., *Sargassum* spp., and *Ecklonia radiata* [89]. Additionally, Fucales, a type of large brown seaweed, such as *Scytothalia dorycarpa*, *Cystophora subfarcinata*, and *Sargassum linearifolium*, are also utilized for their nutritional value and as sources of alginates [89].

The study of Benslima et al. [90] investigated the brown seaweed *Cystoseira schiffneri* as a source of sodium alginate, making a biochemical profile of the extracts. Sodium alginates were derived from *C. schiffneri* harvested from Kerkennah, Sfax, a Tunisian island, during different seasons (December, April, July, and September). The characterization of *C. schiffneri* sodium alginates (CSSA) involved the assessment of their structural features and antioxidant properties. Microelementary analysis revealed the absence of nitrogen and sulphur in the alginate fractions. The alginate isolates exhibited high contents of uronic acids (ranging from 47.4% to 66.4%) and ash (ranging from 24.3% to 39.4%). ATR-FTIR, NMR, and circular dichroism analyses indicated that all CSSA samples were classified as polyguluronate-type. The average molecular weight, determined through high-performance size exclusion chromatography, displayed variations spanning from 4.49 to 1230 kDa. The antioxidant activity of CSSA from different seasons was evaluated using assays for DPPH• radical scavenging, reducing power, and Fe^2+^ chelating ability. The antioxidant potential of CSSA exhibited notable variations depending on the season, with the primary factor governing these properties being the molecular mass [90].

According to Zhu et al. [91], the CSSA fractions demonstrated greater antiradical activity compared to alginate hydrolysates (IC_50_ = 10.4 mg/mL). However, Hentati et al. [92] reported that *Cystoseira compressa* sodium alginate with a molecular weight of 1.11 g/mol exhibited higher activity (IC_50_ = 560 μg/mL) than the samples collected in December, April, and July, although it remained lower than that of the September sample. A positive correlation was observed between radical-scavenging activity at 1500 μg/mL and molecular weight. Thus, the molecular weight appears to be the primary factor influencing the antioxidant properties of sodium alginates. Kelishomi et al. [93] also demonstrated that the radical-scavenging activity of alginates was inversely proportional to the molecular weight. Polysaccharides with higher molecular weights exhibited a more compact structure, leading to stronger intramolecular hydrogen bonds that restrict the accessibility of hydrogen groups to oxidant molecules. As a result, the active groups have a more challenging time interacting with oxidant molecules [90]. In a previous study, the water-soluble alginate derived from *Bifurcaria bifurcata* was examined [94]. NMR analysis of the sodium alginate revealed a higher presence of guluronic acid, particularly in the form of homoguluronic blocks. When compared to alginates from other brown algae, the sodium alginate extracted from *B. bifurcata* with a molecular weight ≤ 5.5 kDa exhibited a greater abundance of guluronic acid compared to alginates from *Cystoseira* species [92,95], *Fucus vesiculosus* [96], *Sargassum species* [97,98,99], *Laminaria japonica* and *Laminaria digitata* [100]. Similar results were observed in alginates from *Cystoseira myrica* and *Laminaria hyperborea* [101]. However, certain *Sargassum* species, notably *Sargassum polycystum* and *Sargassum filipendula*, displayed a higher concentration of guluronic acid [98]. The content of uronic acids and the proportions of homoguluronic blocks play a significant role in determining the physicochemical properties of alginate [102]. Alginate with an M/G (mannuronic acid/guluronic acid) ratio of less than one forms strong and rigid gels [103]. Based on these findings and our own results (M/G < 1 and FGG = 0.45), we can confirm that a gel can be formed using sodium alginate extracted from *B. bifurcata* [104].

## 3. Physical-Chemical Properties of Algal Phycocolloids

When evaluating the quality of an optimal material for the preparation of scaffolds or hydrogels, it is essential to consider mechanical properties such as tensile strength, elongation at break, heat resistance, and water vapour permeability. Tensile strength refers to the maximum stress a material can withstand before breaking when stretched or subjected to pressure. Generally, the tensile strength and Young’s modulus of plant fibres increase with higher cellulose content. Elongation at break is the ratio between the modified length and the initial length of the tested material after it fractures. It indicates the material’s ability to undergo shape changes without developing cracks in a matrix composed of natural polymers. Thermal resistance is a thermal property that represents the temperature difference a material can withstand [105].

Vapour permeability, on the other hand, pertains to a material’s ability to allow the passage of vapours, such as water vapour or other gases. According to ISO 11092:1993 [106], water vapour permeability is a characteristic influenced by the water vapour resistance of a textile material or composite. A higher permeability value indicates that water and vapour can pass through the material more quickly, which is desirable.

The gelling properties of phycocolloids extracted from seaweeds vary depending on factors such as their structure, concentration in a solution, temperature, pH, and syneresis potential [107,108,109]. The physicochemical attributes of phycocolloids (Table 1) are extensively influenced by factors such as species, environmental circumstances, extraction methodologies, and treatment procedures [107,110]. Therefore, it is crucial to possess a comprehensive understanding of these factors to discover the most effective techniques for obtaining high-quality phycocolloids with optimal mechanical properties.

Carrageenans, in their salt form, exhibit significant gel strength [111]. Among them, κ-carrageenan and ι-carrageenan gels remain stable at room temperature, while λ-carrageenan, being the only carrageenan soluble in cold water in its natural state, does not gel. The addition of cations enhances gel formation and strength in κ-carrageenan phycocolloids, as reported by Robal et al. [112]. In a study by Paula et al. [113] the physical properties of glycerol-plasticized edible films made with κ-carrageenan, ι-carrageenan, and alginate were examined. It was found that κ-carrageenan films exhibited higher tensile strength, elasticity, moisture permeability, and lower opacity compared to ι-carrageenan films, whereas alginate films displayed higher transparency [113].

Similar to carrageenan, alginate can form a robust and stable gel structure when exposed to cations, especially Ca^2+^ [114]. Films made from sodium alginate using a 1–3% (*w*/*v*) calcium chloride solution exhibited improved tensile strength and elongation properties, while also displaying reduced opacity [115,116].

Agar films exhibit lower tensile strength and water vapour permeability compared to carrageenan and alginate films. Nevertheless, they demonstrate approximately double the elongation value compared to κ-carrageenan films and exhibit superior elasticity [117,118]. The gelling properties and viscosity of seaweed phycocolloids render them extensively employed as stabilizers, thickening agents, and gelling agents in the manufacturing of food, pharmaceuticals, and beauty care products.

With advancements in methodologies, researchers have been able to study the behaviour of these seaweed-derived compounds when incorporated into biofilms, making them potential candidates for biofilm and packaging development [119,120,121,122]. The method used for film formation has a significant impact on the physical properties and microstructures of the films. For instance, Li et al. [123] conducted a study on chitosan-alginate films prepared through various methods. Their findings revealed that biofilms fabricated through layer-by-layer assembly combined with ferulic acid crosslinking exhibited improved mechanical properties, opacity, and hydrophobicity compared to films prepared through direct mixing, crosslinking alone, and layer-by-layer assembly alone.

When it comes to extraction, carrageenan and agar are typically obtained using hot water as a solvent due to their high solubility. Conversely, the extraction of alginate requires hot alkali as the primary solvent since alginic acid is composed of salts that are insoluble in water. Through alkali extraction, these alginic acid salts are transformed into water-soluble alginate salts. Although hot water extraction is optimal for carrageenan and agar extraction, alkali extraction is commonly used in the industry to preserve their rheological properties, such as gel strength.

Considering these characteristics, seaweed phycocolloids hold potential for incorporation into biofilms or the development of new supports for drug delivery [119,120,121,122].

## 4. Therapeutic Applications of Phycocolloids

Over the past few decades, extensive research has been conducted to investigate the biological capabilities of seaweed phycocolloids (Table 2). Numerous interesting biological properties are exhibited by these natural compounds. Understanding the biological properties of seaweed phycocolloids will help to develop new natural treatments for specific diseases.

### 4.1. Carraagenan

Studies have provided evidence that carrageenans possess interesting biological activities [57,58,143,144,145].

Polysaccharides characterized by the highest degree of sulfation and molecular weight, as well as the most notable in vitro antioxidant capacity, were obtained by sequentially extracting carrageenan from *Mastocarpus stellatus* using water, acid, and alkali [124]. Several studies have reported the enhanced activity of antioxidant enzymes, including catalase and superoxide, in the presence of carrageenans [146]. For example, in vitro systems have shown that κ-carrageenan oligosaccharides derived from *Kappaphycus striatus*, as well as their oversulphated, acetylated, and phosphorylated derivatives, possess antioxidant properties [147]. Furthermore, λ-carrageenan from *Chondracanthus acicularis* and *G. pistillata*, κ-carrageenan from *K. alvarezii*, and ι-carrageenan from *Eucheuma denticulatum* (Sigma-Aldrich) exhibited the most significant hydroxyl radical activity, with an IC_50_ value of 0.281 ± 0.072 μg/mL. Notably, ι-carrageenan demonstrated a more potent inhibition of hydroxyl radicals compared to λ-carrageenan (EC_50_ = 0.357 ± 0.120 μg/mL) and κ-carrageenan (EC_50_ = 0.335 ± 0.016 μg/mL) [39].

Carrageenan has been demonstrated to induce apoptosis in various cancer cell lines. In particular, the red alga *Porphyra yezoensis* has shown the ability to induce dose-dependent cancer cell death through apoptosis in in vitro tumour cell lines, while not exhibiting cytotoxicity against healthy cells [125]. In the same study, the ability of carrageenans derived from *K. alvarezii* to reduce the growth of liver, colon, breast, and osteosarcoma cell lines was detected. Additionally, several studies have reported the antiproliferative effects of carrageenans in in vitro cancer cell lines, as well as the inhibition of tumour growth in mice [126,127]. Carrageenans have also exhibited antimetastatic effects by impeding the interaction of cancer cells with the basement membrane, inhibiting tumour cell growth, restraining tumour cell growth, and preventing adhesion to different substrates. However, the exact mechanisms of action remain unidentified. Furthermore, Yamamoto et al. [148] observed a significant reduction in the incidence of carcinogenesis in vivo through the oral administration of various seaweeds. In the evaluation of colonic carcinogenesis in male rats, carrageenan did not cause any treatment-induced alterations in clinical manifestations or body mass [149].

Carrageenan has exhibited selective inhibition against various human pathogens, including the human immunodeficiency virus (HIV), herpes simplex virus (HSV), human cytomegalovirus, and human rhinoviruses [128]. Its mechanism of action involves blocking the attachment and entry of virions into cells [150]. The resemblance of carrageenan to heparan sulphate, a cell-attachment factor for HPV, supports this conclusion. In vitro studies have shown that carrageenan, as an extract from seaweed, is highly effective in inhibiting HPV infectivity. Additionally, certain strains of HIV1 and herpes simplex viruses have also been found to be susceptible to carrageenan in vitro [129].

In the beginning, λ-carrageenan obtained from the red algae *Gelidium cartilagenium* exhibited antiviral properties against the influenza B virus or mumps virus [130]. The antiviral activity of carrageenan is attributed to its capacity to shield specific cellular structures involved in virus–receptor interaction [151]. The antiviral effect of λ-carrageenan can be attributed to the formation of enduring virion–carrageenan complexes that are irreversible, thus occupying the viral envelope sites necessary for virus attachment to host cells and hindering the virus from completing the infectious cycle [152,153].

Studies have provided evidence of carrageenan antidiabetic properties [154]. In a clinical experiment, individuals who consumed porridge containing λ-carrageenan exhibited significantly lower postprandial glycemic responses compared to controls. This suggests that carrageenan, along with dietary fibre, can contribute to the management of metabolic disorders such as diabetes [131]. Additionally, a separate study explored the antidiabetic activity of carrageenan derived from *Eucheuma cottonii*. The findings demonstrated significantly reduced blood glucose and insulin responses, leading to a 50% reduction in glucose absorption balance in pigs [132,133].

Carrageenan exhibits the ability to prevent infection caused by various microbes. The antimicrobial efficacy of carrageenan was assessed by determining the diameter of the inhibitory zone when using κ-carrageenan oligosaccharides against *Escherichia coli*, *Staphylococcus aureus*, *Saccharomyces cerevisiae*, *Penicillium citrinum*, and *Mucor* spp. The results showed that all κ-carrageenan oligosaccharides exhibited inhibitory effects against the tested bacteria, with *Saccharomyces cerevisiae* displaying the highest activity [134]. Furthermore, when carrageenan was modified as an antimicrobial agent (oxidized κ-carrageenan), it caused damage to the bacterial cell wall and cytoplasmic membrane, resulting in the suppression of growth in both Gram-positive and Gram-negative bacteria. The antibacterial activity of oxidized κ-carrageenan was broad-spectrum, suggesting its potential as a promising candidate for the development of novel antibacterial agents [134].

Azizi et al. developed hydrogel beads by crosslinking κ-carrageenan with biosynthetic silver nanoparticles (Ag-NPs) to form a carrageenan/silver nanoparticle hydrogel composite. In comparison to a pure κ-carrageenan hydrogel, the resultant biologically derived nanocomposite hydrogel exhibited decreased swelling characteristics. Moreover, it demonstrated significant antibacterial efficacy against *S. aureus*, methicillin-resistant *S. aureus* (MRSA), *Pseudomonas aeruginosa*, and *E. coli*, with a maximum inhibitory zone diameter of 11 mm. The cytotoxicity assessment indicated that the nontoxic biological nanocomposite hydrogel exhibited promising pharmacological potential at concentrations below 1000 µg/mL [135].

### 4.2. Alginate

Alginate hydrogels have also gained recognition for their various benefits in wound healing, including reduced scarring, minimal bacterial infection, promotion of cell proliferation, enhancement of cytokine activity, regulation of pain and inflammation, and creation of a moist wound environment [42,155]. These hydrogels have also shown promise in tissue regeneration and drug delivery, as they possess a structural resemblance to the extracellular matrix, allowing them to perform critical functions in wound management [156]. Dry films composed of alginate-based dressings are well suited for the treatment of superficial wounds that exude fluid. When an alginate-based dressing is applied to a wound with a moderate to high level of exudate, the alginate component within the dressing absorbs the fluid, effectively preventing maceration of the surrounding tissues and promoting wound healing [41]. Upon the application of the alginate film, the dressing with divalent calcium ions is released and replaced by monovalent sodium ions present in the wound bed or exudate. This process leads to the formation of hydrophilic gels [157]. Through ion exchange and fluid absorption from the exuding wound, the alginate film gradually transforms into a gel, providing a moist environment while also reducing bacterial infections and facilitating processes such as cell proliferation, migration, and epithelialization. Over time, the alginate film gradually dissolves, further aiding the healing process [158].

Alginate, a biomaterial that forms a gel upon the introduction of divalent calcium ions (Ca^2+^), has been extensively investigated and employed in diverse domains such as wound healing, tissue engineering [159], orthopaedics, and dental implant surgery. Its key benefits include low toxicity, cost-effectiveness, biocompatibility, and osteoconductive [160,161].

The incorporation of ceramics into alginate composite materials plays a significant role in enhancing the mechanical strength of the extracellular matrix. In a study conducted by Zhao et al. [136], an injectable hydrogel composed of calcium phosphate alginate and umbilical cord mesenchymal stem cells (UCMSCs) exhibited significant osteogenic differentiation. This was evidenced by elevated alkaline phosphatase activity (ALP), the expression of osteocalcin (OC), collagen I, and mineralization [136]. Bouhadir et al. [137] introduced an alginate hydrogel containing chondrocytes, responsible for maintaining the extracellular matrix and producing the cartilage matrix, into the dorsal region of mice. After 7 weeks, the hydrogel-containing chondrocyte scaffolds were removed, revealing a white opalescence indicating the presence of native cartilage. This was confirmed through standard trichrome blue staining. In contrast, unmodified alginate and chondrocytes resulted in small cartilage-like tissues surrounded by many alginate residues. The weight reduction of the hydrogel implant within the 7-week period was attributed to the degradation of the hydrogel and faster release of oxidized alginate compared to in vitro studies. This injectable scaffold shows promise in the regeneration of cartilage-like tissues [137]. Moshaverinia et al. [138] developed an injectable and biodegradable system using oxidized alginate microbeads to encapsulate periodontal ligament and gingival mesenchymal stem cells (GMSCs). In vitro studies demonstrated a high level of osteodifferentiation and adipodifferentiation in this system, suggesting its potential in periodontal tissue regeneration [138].

Alginate hydrogels are utilized in the field of wound healing for the development of wound dressings [162,163,164,165]. Numerous studies have demonstrated that drugs encapsulated within alginate hydrogels exhibit improved bioavailability compared to the free form of the drug directly applied to the wound site, leading to enhanced healing efficacy. Moreover, alginate hydrogels find extensive applications in tissue regeneration and cell encapsulation therapies [166,167,168,169,170,171,172]. Alginate is commonly employed in the fabrication of capsules for cell encapsulation, which is often associated with cytotherapy treatments or the creation of cellular microcultures within more complex systems. A novel approach to constructing alginate-based capsules has been proposed, wherein cells are encapsulated in liquefied alginate particles and subsequently coated with chitosan and alginate. Additionally, coencapsulation of cells with poly(lactic acid) microparticles provides protection and ensures high cell viability within the encapsulated system.

Alginate-derived hydrogels currently offer several advantages, making them suitable materials for applications in tissue engineering and regenerative medicine [173,174,175]. The ionotropic alginate hydrogel biomaterial has gained significant attention in the fields of tissue engineering and regenerative medicine due to its biocompatibility, non-thrombogenic nature, mild gelation process, and similarity to the extracellular matrix (ECM). As a result, it has been widely utilized as a drug delivery system in these domains [176]. The study of Freeman et al. [177] have provided evidence that the implantation of sodium alginate hydrogel does not result in harm to the healthy myocardium and, instead, contributes to an increase in scar thickness due to its provision of physical support.

In various studies, sodium alginate hydrogel has been utilized as a carrier for the controlled release of various bioactive cytokines, with the goal of promoting self-healing and facilitating natural regeneration [178,179]. In the context of treating myocardial infarction (MI), small extracellular vesicles (sEVs) derived from bone marrow mesenchymal stem cells (MSCs) have shown promise. However, challenges such as low retention and short-lived therapeutic effects persist. The study conducted by Lv et al. [139] aimed to investigate whether incorporating MSC-derived sEVs into alginate hydrogel could improve their retention in the heart and subsequently enhance the therapeutic effects. The researchers determined the optimal formulation of sodium alginate hydrogel incorporating sEVs based on their release profile and the rheological properties of the hydrogel. Ex vivo fluorescence imaging was employed to assess the retention of sEVs in the heart, while immunofluorescence staining was used to analyse immunoregulation and the effects of sEVs on angiogenesis. Cardiac function and infarct size were estimated using echocardiography and Masson’s trichrome staining, respectively. The results revealed that the delivery of sEVs incorporated in alginate hydrogel (sEVs-Gel) improved their retention in the heart. Treatment with sEVs-Gel demonstrated a significant reduction in cardiac cell apoptosis and promoted macrophage polarization at day three post-MI, compared to the treatment with sEVs alone (sEVs). Furthermore, sEVs-Gel treatment led to increased scar thickness and angiogenesis four weeks after the infarction.

Cardiac function and infarct size measurements also showed significant improvements in the sEVs-Gel group compared to the group treated with sEVs alone. Overall, the delivery of sEVs incorporated in alginate hydrogel presents a novel approach to cardiac therapies [139]. Various studies have highlighted the positive attributes of alginic acid and its derivatives, including biocompatibility, antioxidant properties, and anti-inflammatory effects [180,181,182,183]. Currently, these compounds find widespread use in the cosmetic industry, particularly in products targeting antiwrinkle, moisturizing, and sun protection effects [184]. However, their application in drug delivery systems has gained attention more recently, showcasing significant potential for various applications. This emerging field holds promise and is expected to shape future advancements and directions in the field.

### 4.3. Agar

Agar is a colloidal substance extracted from various red algae species that has gained widespread use in several sectors regarding biomedicine, chemicals, and the alimentary services, primarily as a gel-forming agent, thickener, water-holding agent, and stabilizer. The properties of agar, including its affordability, thermoreversibility, and high-strength nature, contribute to its increasing utilization across various industries. The chemical composition of agar, such as its sulphate, methoxyl, and sugar contents, influences its application in different products, and this composition can be affected by various extraction process variables. With its remarkable gelling power and the ease of extraction, agar holds promise as an environmentally friendly material due to its renewability and biodegradability. While previous research has focused on utilizing agar or alginate alone for oral drug delivery, there is limited exploration of blending agar with natural or synthetic polymers to modify its properties [185].

Ninan et al. [140] developed an innovative approach to fabricate carboxylated agarose/tannic acid hydrogel scaffolds cross-linked with zinc ions, enabling pH-controlled release of tannic acid. These hydrogels demonstrated minimal tannic acid release under neutral and alkaline pH conditions, while exhibiting sustained release at acidic pH, accompanied by maximum swelling. Furthermore, the hydrogels exhibited favourable antibacterial activity and demonstrated no cytotoxic effects on 3T3 fibroblast cell lines. In simulated wound assays, cells exposed to tannic acid hydrogel extracts exhibited significantly enhanced migration and proliferation. Additionally, the tannic acid hydrogels effectively suppressed the production of nitric oxide (NO) in stimulated human macrophages in a concentration-dependent manner, indicating potent anti-inflammatory activity. These findings highlight the potential of carboxylated agarose/tannic acid hydrogel scaffolds as promising candidates for wound dressings.

Agarose solutions exhibit the ability to form reversible gels upon cooling below 40 °C through physical cross-linking [74]. This property, along with its unique mechanical characteristics and biocompatibility, has made agarose widely used in various applications such as cosmetics, biomedical applications, cell therapies, drug delivery, tissue engineering, and molecular biology. While native agarose hydrogels are generally not suitable for cell adhesion and growth due to their high hydrophilicity and antiadhesive properties, this feature is advantageous in hydrogel dressings. It prevents tissue ingrowth into the matrix, minimizing potential damage to the healing wound upon removal [140]. Agar is an FDA-approved biocompatible natural substance, and it has found extensive use as both a food additive and a biomedical device. It possesses properties such as the ability to protect cells from oxidative stress caused by active oxygen and exhibits unique mechanical characteristics, especially shear-thinning behaviour in viscous systems [141,186].

In their study, Baek et al. [142] investigated the effects of agar on gellan gum (GG) solutions to enhance their injectability while preserving their mechanical and biological properties. GG is commonly used in the food industry and has been suggested for various biomedical applications. This polysaccharide exhibits robust mechanical, chemical, and physical resilience when crosslinked, making it compatible for implantation in diverse tissues [187]. However, the viscosity of cross-linked GG poses a challenge for in-situ injection. To address this issue, additional compounds can be incorporated into the GG solution to act as thinners and enhance injectability. The researchers mixed different concentrations of agar with GG and crosslinked the mixture using calcium chloride. Subsequently, they evaluated the injectability of each hydrogel. Through this process, they synthesized various microporous scaffolds and conducted mechanical, chemical, and biological assessments to determine the suitability of GG/Agar hydrogels as carriers for cell-based cartilage regeneration [142].

The inclusion of agar in the hydrogel led to notable improvements in mechanical properties, including increased compressive strength and shear stress, which can be attributed to the higher Young’s modulus of the added compound. Importantly, the addition of agar enabled injectability of the hydrogel through a syringe needle, overcoming a significant limitation of using GG as a biomedical device. The morphology of the materials exhibited an appropriate porous microstructure, with a porosity range of 70 to 180 µm, facilitating optimal water uptake for improved mechanical properties, as well as effective diffusion of nutrients, oxygen, and cell ingrowth. Furthermore, the presence of agar was found to promote higher cell proliferation rates and increased cell survival, indicating its positive impact on chondrocyte growth and viability. Incorporating different concentrations of agar demonstrated the ability to enhance both the desirable biological and mechanical properties of GG. Among all the scaffolds tested, the 0.8 wt% GG/agar formulation showed the most promising potential for cartilage regeneration. Additionally, the injectability of the material allows for in-situ application, enabling minimally invasive procedures for cartilage tissue repair [142].

## 5. Phycocolloids as Potential Drug Delivery System

The common employed practice for regulating the release of drugs is through the utilization of oral extended-release tablets consisting of a solitary hydrophilic matrix system. The properties of carrageenan, such as its elevated molecular weight, high viscosity, and gelling capabilities, make it a suitable choice for serving as the matrix in the creation of extended-release tablets. However, its capacity for loading drugs is limited. In order to surmount this constraint, the combination of polymers has been extensively employed over many years to effectively adjust drug release and achieve desirable outcomes [188].

The impact of polymer blends that include carrageenans (τ, ʎ) and cellulose ethers (HPMC, sodium carboxymethylcellulose, methylcellulose, hydroxypropyl cellulose) on the release of ibuprofen from tablets, produced through direct compression, was examined. Most of the formulations displayed consistent release patterns, and the release of ibuprofen was sustained for a duration of 12–16 h [189]. Additionally, carrageenan, when combined with various polymers, can be employed to develop three-layered matrix tablets. In comparison to HPMC, pectin, guar gum, xanthan gum, chitosan, and ethyl cellulose, carrageenan has been recognized as the most effective polymer for drug delivery [190]. Moreover, carrageenan, being negatively charged above its pKa value, can spontaneously interact with positively charged polyions to form polyelectrolyte complexes [191].

The erosion of matrix tablets based on ʎ-carrageenan can be influenced by the pH of the dissolution media. However, incorporating a less rapidly degradable hydroxypropyl methylcellulose (HPMC) into the ʎ-carrageenan matrix helps to minimize the disparity in drug release across dissolution media with varying pH [192]. Furthermore, by optimizing the ʎ-carrageenan/HPMC ratio in the matrix [193], a linear and pH-independent release profile of chlorpheniramine maleate lasting for 24 h was achieved.

Carrageenan has demonstrated its ability to regulate early drug dumping, making it a promising option for achieving consistent drug release. A study revealed that incorporating as much as 40% ʎ-carrageenan in the matrix (ʎ-carrageenan-HPMC) effectively controlled the initial release of drugs, such as salbutamol sulphate and chlorpheniramine maleate, with release profiles closely resembling linearity [192]. The mechanisms responsible for the reduction in burst release were explained by the interaction between the basic drug and ʎ-carrageenan at the surface of the tablet, which hindered drug diffusion, while the main determinant of drug release was the erosion of the carriers [194]. The combined effect of water uptake, erosion, and interaction may result in prolonged drug release exceeding 24 h [195].

Chitosan is a cationic polymer derived from deacetylation of chitin, consisting of (1-4)-2-amino-2-deoxy-β-D-glucan. It possesses favourable processability and reactivity due to the presence of free hydroxyl and amino groups in its structure [196]. With its high hydrophilicity and semicrystalline nature, chitosan exhibits hydrogen bonding with water molecules, unlike many hydrocolloids that have anionic properties in aqueous environments. When chitosan is dissolved in acidic solutions, it becomes cationic and soluble. This leads to a strong attraction between chitosan and these hydrocolloids when they are blended together [197]. Chitosan has diverse biological activities and finds applications in the food industry and medical fields, especially in drug delivery and wound healing [198,199,200,201].

The chitosan carrageenan and gelatin (CCG) scaffold have been shown to provide excellent support for the attachment and proliferation of adipose-derived mesenchymal stem cells (ADMSC). Additionally, it enhances the osteogenic differentiation and neovascularization capacities of ADMSC. Li et al. [202] developed an iota-carrageenan/chitosan/gelatin (CCG) scaffold using ion shielding technology and an ultrasonic dispersion method to promote the in vitro osteogenic differentiation of adipose-derived MSCs. Tapia and colleagues utilized polyelectrolyte complexes of chitosan and κ-carrageenan as matrices for prolonged drug release. However, the high water absorption capacity of κ-carrageenan in the tablets led to premature disintegration instead of matrix swelling [203,204]. Studies have demonstrated that when tablets based on chitosan-carrageenan are transferred from simulated gastric fluid to simulated intestinal fluid, an in situ polyelectrolyte film can form on the tablet surface, which further regulates drug release [205,206].

Furthermore, the results indicated that chitosan-λ-carrageenan-based matrices exhibited promise as controlled-release drug carriers due to their lower sensitivity to pH and ionic strength compared to chitosan-κ-carrageenan and chitosan-ι-carrageenan matrices [206]. Carrageenan maintains a certain degree of ionization even at low pH due to its low pKa value [207], making it suitable for the preparation of gastric floating tablets [208].

According to Volod’ko et al. [209], carrageenan, characterized by a high degree of sulphation and a flexible macromolecular structure, displays the strongest binding affinity for chitosan. The type of carrageenan used significantly influences the formation of the complex. In another investigation conducted by Volod’ko et al. [210], soluble polyelectrolyte complexes comprising chitosan and carrageenan were developed. These complexes exhibited gastroprotective effects by forming a protective layer on the stomach’s surface (mucosa). This layer acted as a barrier, preventing direct interaction with ulcerogenic agents such as indomethacin.

Grenha et al. [211] have reported on the utilization of carrageenan-chitosan nanoparticles for drug release. The nanoparticles, synthesized using fully hydrophilic conditions, exhibited prolonged release of the macromolecule ovalbumin. The low toxicity observed on fibroblast-like cells indicated the biocompatibility and safety of this nano-carrier, suggesting its potential as a drug delivery system in tissue engineering applications.

Hydrogel beads composed of grafted polyacrylamide with κ-carrageenan were designed to deliver the non-steroidal anti-inflammatory drug ketoprofen [212] to the small intestine. Over a 14-day culture period, human adipose-derived stem cells (hASCs) demonstrated the expression of specific cartilage extracellular matrix molecules, indicating that κ-carrageenan is a viable biomaterial for the encapsulation of cells and growth factors [213].

Matrices composed of ι-carrageenan and λ-carrageenan demonstrated the sustained release of different drug models, and the release profiles exhibited zero-order kinetics. The diameter of the matrix, the ratio of drug to carrageenan, and the ionic strength of the dissolution medium all contributed to the drug release from these matrices [214].

Both κ-carrageenan and alginate are polyelectrolytes that tend to form physical hydrogels when combined with poly and monovalent metal cations.

Alginate is commonly utilized for immediate drug release and sustained drug release for rapid absorption, offering reproducible and predictable kinetics of drug release, respectively. Sodium alginate has found extensive application as a binder in tablet formulation, whereas alginic acid is employed as a disintegrant in compressed tablets to achieve immediate drug delivery [215].

Several researchers have published review articles discussing the use of alginate for drug release [3,216,217,218,219]. Alginate serves as a versatile matrix for the attachment and delivery of biomolecules, including DNA, proteins, and cells. The gelation process, which does not involve the use of organic solvents, facilitates the introduction of biomolecules and cells into the matrices without altering their three-dimensional structure [220].

Alginate has also been utilized in the fabrication of gel capsules. Additionally, it has been used as a supplementary material in the production of 3D porous scaffolds made from β-tricalcium phosphate, demonstrating improved cell seeding and efficient controlled release of growth factors, making it suitable for use in bone tissue engineering [221].

Alginates have the ability to create robust complexes with other natural polyelectrolytes, such as pectin (also a polyuronate), through chain–chain associations and the formation of hydrogels upon the addition of divalent cations (e.g., Ca^2+^) [222].

This interaction boosts encapsulation effectiveness and enhances the chemical and mechanical durability of alginate beads. Alginates are commonly used to produce biopolymer films due to their colloidal properties. Films formed solely by κ-carrageenan may have inferior quality, but the presence of numerous sulfonic groups in κ-carrageenan makes it an attractive material for enhancing proton exchange membranes (PEMs).

Nevertheless, the high solubility in water and inadequate mechanical characteristics present obstacles for these polymeric membranes. Crosslinking of the polymer matrix is essential to reduce solubility in water and improve mechanical properties [223,224]. Various functional groups present in these polysaccharides, such as -COOH or -OH groups, allow reactions with ions (K^+^, Ca^2+^), diimine, glutaraldehyde (GTA), and others to produce crosslinked films [225].

**Figure 4:**
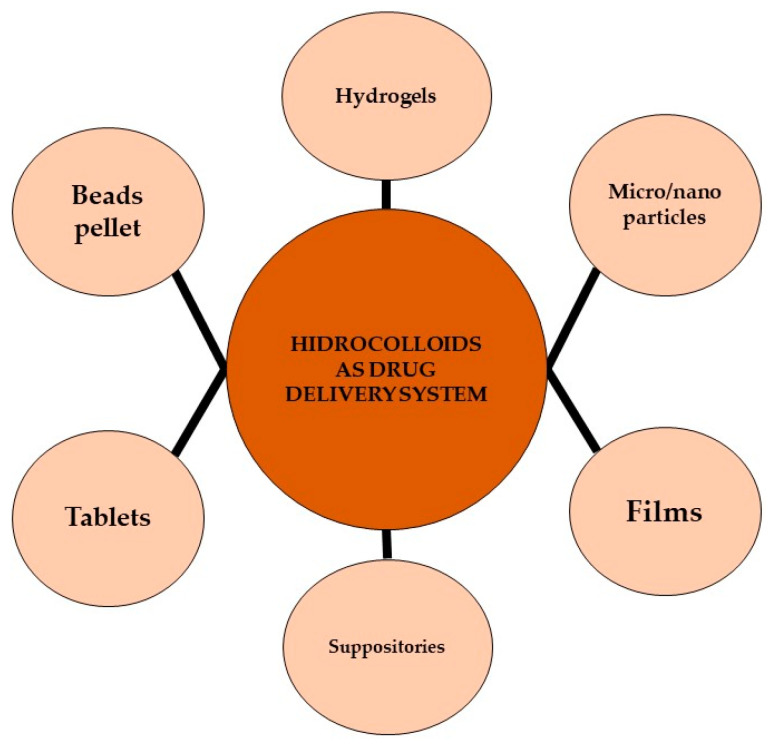
Schematic description of drug delivery system components that might be developed with seaweed hydrocolloids.

Hydrocolloids can be applied in various pharmaceutical formulations, including tablets, suppositories, films, beads, pellets, microparticles, nanoparticles, and hydrogels (Figure 4).

Cabello et al. [226] fabricated biopolymeric membranes composed of κ-carrageenan and alginate at varying ratios for the application of low-temperature direct methanol fuel cells (DMFC). The results demonstrated that increasing the carrageenan content in the membranes also increased the methanol permeability, making the bio-polymeric membranes suitable for application as polymer electrolyte membranes in DMFCs.

Pascalau et al. [227] prepared crosslinked composite films of alginate/κ-carrageenan using CaCl_2_, demonstrating enhanced swelling behaviour and superior mechanical properties when compared to pure κ-carrageenan and alginate films. These composite films hold potential for biomedical applications.

Alginate fibres are frequently generated by immersing a solution of water-soluble alginate, commonly sodium alginate, into a bath comprising either a calcium salt solution or an acidic solution. This process results in the formation of alginate calcium fibres or acid alginate fibres, respectively. These fibres have the potential to be utilized in the production of fabrics and yarns for medical purposes [228,229].

In a study by Fan et al. [230], a novel blend of fibres was prepared by spinning a mixture solution of carboxymethyl κ-carrageenan and alginate through a viscose-type spinneret into a coagulating bath consisting of aqueous ethanol and CaCl_2_. The combination of these substances enhanced their thermal resistance and water retention characteristics. Moreover, upon treatment with an aqueous solution of silver nitrate, the fibres exhibited antimicrobial properties with effective activity against *S. aureus* were obtained. These unique blend fibres, comprising carboxymethyl κ-carrageenan and alginate, hold great potential for applications in wound healing dressings [230]. The utilization of κ-carrageenan in wound healing and dressing is presently constrained. Nevertheless, initial laboratory investigations on hydrogels made from poly(N-vinyl-2-pyrrolidone)-κ-carrageenan have shown promising characteristics for wound dressing applications. These hydrogels display favourable attributes such as robust mechanical strength, transparency, biocompatibility, prevention of bacterial infection, regulation of evaporative water loss from the wound surface, and permeability to gases. These characteristics are crucial requirements for effective wound dressing materials [231].

**Figure 5:**
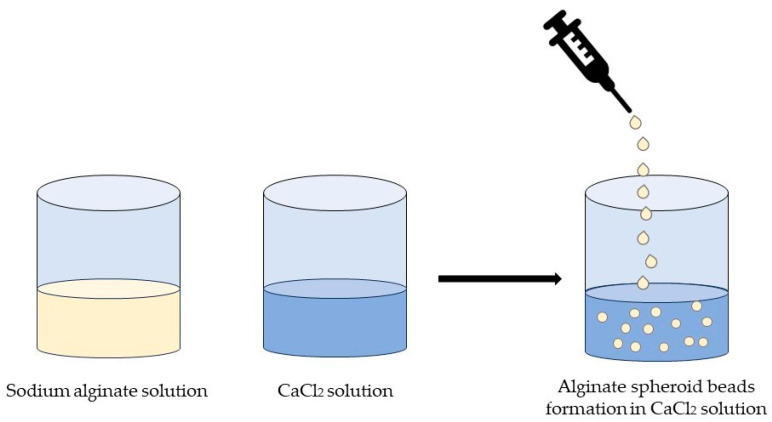
Schematic illustration of alginate beads formation.

Sodium alginate, on the other hand, can rapidly form a gel under very mild conditions [232,233]. The combination of sodium alginate and calcium ions forms gel beads that possess freeze resistance and can be rehydrated after drying (Figure 5). The strength of the gel is directly affected by the concentrations of calcium ions and sodium alginate, where higher concentrations lead to stronger gels. The gelation process can be managed by pH adjustment, the selection of suitable calcium salt, the incorporation of a phosphate buffer, or the utilization of a chelating agent [234]. The viscosity of sodium alginate is closely associated with the brittleness of the gel, with higher viscosity resulting in increased brittleness. However, the rigidity of the gel can be modified by adjusting the ratio of sodium alginate to acid [235].

In addition, the temperature-responsive properties of alginate gels offer a wide range of potential applications as smart drug carriers. Durkut et al. [236] developed a thermosensitive polymer known as poly(N-vinylcaprolactam)-grafted aminated alginate (PNVCL-g-Alg-NH_2_), which underwent a phase transition and expansion upon water absorption at approximately 35 °C, making it suitable for physiological temperature conditions. Moreover, copolymerization with PNVCL reduced the water absorption of aminated alginate and enhanced its thermal stability. Biocompatibility studies, including in vitro cytotoxicity and blood compatibility analyses, confirmed the nontoxicity of PNVCL-g-Alg-NH_2_ scaffolds and their lack of haemolytic effects [236].

Liu et al. [237] synthesized a thermally responsive copolymer, alginate-g-PNIPAAm, by coupling poly(N-isopropylacrylamide) (PNIPAAm) with sodium alginate. The copolymer could be dissolved in water or a phosphate-buffered saline solution at room temperature (25 °C). Upon reaching the critical micelle temperature, the copolymer underwent self-assembly into micelles with a low critical micelle concentration. Subsequently, when the temperature reached body temperature (37 °C), the copolymer underwent a phase transition and transformed into a thermosensitive hydrogel. A practical application of this copolymer involved the construction of an injectable thermally responsive sustained release hydrogel loaded with doxorubicin (DOX), a chemotherapy drug. The hydrogel exhibited a continuous release of DOX-encapsulated micelles, which demonstrated enhanced uptake of multidrug-resistant AT3B-1 cells and effectively induced cancer cell death, thus overcoming drug resistance.

In a recent study conducted by Bagher et al. [238], an alginate-chitosan hydrogel system containing hesperidin for drug release in wound healing was developed. Scanning electron microscopy analysis of the morphology of the hydrogel revealed a highly porous microstructure with interconnected pores. The in vitro release of hesperidin from the alginate-chitosan hydrogel system demonstrated sustained release over a 14-day period. Moreover, the biodegradability of the hydrogel was confirmed by observing a weight loss of approximately 80% after 14 days. In vitro cell growth investigations showed that cells treated with the alginate-chitosan hydrogel containing 10% hesperidin exhibited higher cell proliferation compared to the control. To assess the in vivo wound healing potential, a full-thickness dermal wound model in rats was utilized. The results clearly indicated that the developed alginate-chitosan hydrogel containing 10% hesperidin promoted better wound closure compared to gauze-treated wounds (control). Overall, these findings suggest that the alginate-chitosan hydrogel system containing 10% hesperidin is a promising candidate for the treatment of skin injuries in humans.

In a study conducted by Babavalian et al. [239], synthetic alginate sulphate hydrogels were utilized as chain networks to entrap a macromolecular therapeutic agent, specifically recombinant PDGF-BB. Calcium ions were employed as crosslinkers in these alginate-based hydrogels. The presence of calcium ions within the hydrogels allowed for the exchange with sodium ions, leading to the breakdown of nonspecific bonds. This process potentially resulted in enhanced porosity of the hydrogel, which facilitated the release of the therapeutic agent [239].

The study conducted by Yin et al. [185] aimed to develop agar-alginate composite hydrogel beads with different proportions, crosslinked using Ca^2+^. The chemical composition of the agar-alginate blends and the formation of the crosslinked structure were analysed using FTIR spectroscopy. The surface structure characteristics of the samples were observed using scanning electron microscopy (SEM). The pH-responsive swelling and controlled drug release behaviours of the composite agar-alginate gel samples were evaluated. The inclusion of agar had a significant impact on the swelling and drug release behaviours while retaining the pH-responsiveness properties. The drug release profiles indicated that the presence of calcium chloride prevented drug leakage from the microspheres and reduced the rate of drug release. The incorporation of agar improved the drug loading capacity and mechanical stability of the microspheres. The agar/alginate beads exhibited excellent properties in terms of swelling, drug release, and pH-sensitivity, making them promising candidates for oral drug delivery in the field of biomedicine.

Moreover, gel beads with a higher agar content showed slower rates of swelling and drug release within a 720 min timeframe. Further investigation of the drug release behaviour of the agar/alginate beads was performed in simulated intestinal and gastric fluids, demonstrating their potential as controlled drug delivery systems [185].

## 6. Conclusions

In conclusion, seaweeds continue to be valued for their remarkable benefits. Their composition is characterized by low fat content and an abundance of unique biological compounds that make them promising subjects for biotechnological applications. Hydrocolloids derived from seaweeds have extensive use in several industries as gelling agents, coatings, stabilizers, and cosmetic ingredients. Exploring the beneficial properties of these seaweed compounds opens opportunities for developing specific functional foods and medical products tailored to different needs. Since they demonstrated therapeutical activities, it is highly important to develop new systems to include phycocolloids in the development of new medical products.

There is a consistent literature about the use of carrageenan, alginate, and agar for pharmaceutical purposes and the development of new systems of drug delivery. Moreover, blending carrageenan and alginate with natural polymers holds promise for creating biodegradable materials with safe biomedical applications. Overall, the therapeutic potential of carrageenan, alginate, and agar derived from seaweeds is supported by research, indicating their potential in developing innovative drug delivery systems utilizing these seaweed’s phycocolloids. Further exploration and development in this field hold promise for advancements in various biomedical applications.

## Figures and Tables

**Table 1:** Physical-chemical characteristics of the main seaweeds phycocolloids.

Compound	Physical-Chemical Characteristics
Carrageenan	
κ-carrageenan	High tensile strength, elasticity, moisture permeability, high transparency
ι-carrageenan	Low strength and elasticity, high opacity
λ-carrageenan	Soluble in cold water and does not form gel in its natural state
Alginate	High tensile strength and elongation, high transparency
Agar	Low tensile strength, low water vapour permeability, High elongation and elasticity

**Table 2:** Biological capabilities of seaweed phycocolloids and origin based on literature studies.

Investigated Compound	Origin of the Compound	Therapeutical Activity	References
Carrageenan	*Mastocarpus stellatus*	Antioxidant activity	[124]
κ-carrageenan	*Kappaphycus alvarezii*	Antioxidant activity	[125,126]
λ-carrageenan	*Chondracanthus acicularis* *Gigartina pistillata*	Antioxidant activity	[39]
κ-carrageenan	*Kappaphycus alvarezii*	Antioxidant activity	[39]
ι-carrageenan	*Eucheuma denticulatum*	Antioxidant activity	[39]
Carrageenan	*Porphyra yezoensis*	Anticancer activity	[125]
Carrageenan	*Porphyra yezoensis*	Inhibition of tumour growth in mice	[126,127]
Carrageenan	Purchased	Antiviral activity	[128]
Carrageenan	Purchased	Antiviral activity	[129]
λ-carrageenan	*Gelidium cartilagenium*	Antiviral activity	[130]
λ-carrageenan		Antidiabetic activity in clinical study	[131]
carrageenan	*Eucheuma cottonii*	Antidiabetic activity	[132,133]
κ-carrageenan oligosaccharides	Purchased	Antibacterial activity	[134]
Carrageenan/silver nanoparticle hydrogel composite	Purchased	Antibacterial activity	[135]
Calcium phosphate-alginate hydrogel	Purchased	Osteogenic differentiation	[136]
Alginate hydrogel	Purchased	Cartilage tissues regeneration	[137]
Alginate microbeads	Purchased	Osteodifferentiation and adipodifferentiation	[138]
Alginate hydrogel	Purchased	Increase in scar tissue thickness	[135]
MSC-derived sEVs alginate hydrogel	Purchased	Reduction in cardiac cell apoptosis in mice	[139]
Carboxylated agarose/tannic acid hydrogel	Purchased	Antibacterial and anti-inflammatory activity	[140]
Agarose	Purchased	Antioxidant activity	[141]
Gellan gum/agar hydrogel	Purchased	Cartilage tissues regeneration	[142]

## Data Availability

Not applicable.
